# Metabolic reprogramming of metastatic breast cancer and melanoma by *let-7a* microRNA

**DOI:** 10.18632/oncotarget.3235

**Published:** 2014-12-29

**Authors:** Anastassia Serguienko, Iwona Grad, Anna B. Wennerstrøm, Leonardo A. Meza-Zepeda, Bernd Thiede, Eva W. Stratford, Ola Myklebost, Else Munthe

**Affiliations:** ^1^ Department of Tumor Biology, Institute of Cancer Research, The Norwegian Radium Hospital, Oslo University Hospital, Oslo, Norway; ^2^ Cancer Stem Cell Innovation Centre, Oslo, Norway; ^3^ Genomics Core Facility, Oslo University Hospital, Oslo, Norway; ^4^ The Biotechnology Centre of Oslo, University of Oslo, Oslo, Norway; ^5^ Department of Biosciences, University of Oslo, Oslo, Norway

**Keywords:** ROS, OXPHOS, glycolysis, mitochondria, HMOX1

## Abstract

*Let-7* microRNAs (miRNAs) are highly conserved well-established promoters of terminal differentiation that are expressed in healthy adult tissues and frequently repressed in cancer cells. The tumor suppressive role of *let-7* in a variety of cancers *in vitro* and *in vivo* has been widely documented and prompted these miRNAs to be candidate genes for miRNA replacement therapy. In this study we described a new role of *let-7a* in reprogramming cancer metabolism, recently identified as a new hallmark of cancer. We show that *let-7a* down-regulates key anabolic enzymes and increases both oxidative phosphorylation and glycolysis in triple-negative breast cancer and metastatic melanoma cell lines. Strikingly, the accelerated glycolysis coexists with drastically reduced cancer features. Moreover, *let-7a* causes mitochondrial ROS production concomitant with the up-regulation of oxidative stress responsive genes. To exploit these increased ROS levels for therapeutic purposes, we combined *let-7a* transfection with the chemotherapeutic drug doxorubicin. In both cancer types *let-7a* increased cell sensitivity to doxorubicin. Pre-treatment with N-acetyl cysteine (NAC) totally abolished this effect, indicating that the increased doxorubicin sensitivity of *let-7a* cells depends on the redox pathway. We thus have demonstrated that *let-7a* plays a prominent role in regulating energy metabolism in cancer cells, further expanding its therapeutic potential.

## INTRODUCTION

MicroRNAs (miRNAs) are evolutionarily conserved small (18-25 nucleotides) non-coding RNAs that modulate gene expression by targeting mRNAs of protein-coding genes. *Let-7* miRNA was first identified in *C.elegans* as a heterochronic gene, which promotes larval stage 4-to-adult transition [1]. Further research on *let-7* revealed a highly conserved miRNA family present in vertebrates, ascidians, hemichordates, molluscs, annelids and arthropods [2]. In humans, the *let-7* family consists of 12 members, all sharing a common seed sequence. *Let-7* miRNAs are involved in many physiological, as well as pathological processes, with a primary role in the induction of terminal differentiation and maintenance of this differentiated state throughout lifespan. Many known *let-7* target genes, such as *MYC*, *CCND1*, *RAS*, *LIN28* and *HMGA2* are oncogenes involved in cell cycle progression and stemness. *Let-7* levels were found to be low in a variety of primary and metastatic tumors, and its loss or down-regulation is associated with increased cancer aggressiveness and poor clinical outcome [3-5]. Ectopic expression of *let-7* reduces chemoresistance and invasiveness of cancer cells and suppresses tumor growth of human lung cancers *in vivo* [6].

In recent years reprogrammed metabolism has been recognized as a new hallmark of cancer [7]. The majority of differentiated cells oxidize glucose to carbon dioxide in the mitochondrial tricarboxylic acid (TCA) cycle, generating the amount of ATP necessary to maintain cell homeostasis and to accomplish specialized cellular functions. In contrast, rapidly proliferating cancer cells to meet their metabolic demand activate aerobic glycolysis, a phenomenon known as “the Warburg effect”. During this process a significant part of glucose-derived carbon is diverted into anabolic pathways in order to build up biomass. A modulation of the glucose flux through the glycolytic pathway together with cataplerotic removal of TCA cycle intermediates allow cancer cells to optimize the production of ATP and building blocks for macromolecular synthesis [8]. Oncogenes such MYC and RAS induce the pentose phosphate pathway (PPP), while the tumor suppressor protein TP53 represses PPP by inactivating the rate-limiting enzyme glucose-6-phosphate dehydrogenase (G6PD) [9, 10]. Similarly, fatty acid synthase (FASN), the key enzyme of *de novo* lipogenesis, is found to be highly active in a large variety of cancers, and its up-regulation is associated with chemotherapeutic drug resistance [11, 12]. Thus, counteracting the tumor's anabolic activity may offer a promising therapeutic strategy.

Although in many cancers mitochondria still remain the major source of ATP, the truncation of the TCA cycle caused by cataplerotic reactions or altered mitochondrial biogenesis may decrease the efficiency of mitochondrial oxidative phosphorylation (OXPHOS) [13, 14]. It has been shown that cancer cells with predominantly glycolytic metabolism are more malignant. Cells systematically treated with the mitochondrial inhibitor oligomycin repress OXPHOS and generate larger and more aggressive tumors [15]. One consequence of ongoing OXPHOS is the production of reactive oxygen species (ROS). High level of ROS is harmful for the cells. However, below a toxic threshold, ROS play an essential physiological role as signaling molecules. An increase in ROS levels is required for a variety of stem cells to differentiate and the treatment with exogenous ROS impairs stemness [16-18]. Normal stem cells and cancer stem cells share this property. Indeed, mammary epithelial stem cells and breast cancer stem cells both contain lower ROS level than their more mature progenitors [19]. An association between advanced metastatic state and reduced ROS levels has been shown in breast cancer [20]. Interestingly, a switch from mitochondrial OXPHOS, the major cellular source of ROS, to aerobic glycolysis is also observed during the generation of induced pluripotent stem cells [21]. Taken together these data suggest an inverse association between ROS level and stemness, where a lower level of mitochondrial ROS and reduced mitochondrial activity correspond to a more de-differentiated state. Furthermore, ROS levels have implications for anticancer therapy, although the question is complex. On one hand, increased levels of oxidants likely make cancer cells more vulnerable to further damage by therapy-induced exogenous ROS treatment. On the other hand, a persistent intrinsic oxidative stress causes the up-regulation of ROS scavenging system or employment of a “go or grow” strategy making cancer cells better-adapted and thus more resistant [20, 22].

Recently *let-7* miRNA has been shown to regulate glucose metabolism through m-TOR dependent and m-TOR independent mechanisms *in vitro* and *in vivo* [23, 24]. However, due to the metabolic heterogeneity of cancer in general, in depth study of specific cancer models is needed. In the present study we have addressed the role of *let-7* in regulating energy metabolism in triple-negative breast cancer and metastatic melanoma cell lines, and explored the therapeutic potential of the miRNA replacement therapy *in vitro* in combination with the conventional anticancer drug doxorubicin.

## RESULTS

### *Let-7a* represses proliferation and clonogenic capacity of MDA-MB-231 cells

**Figure 1:**
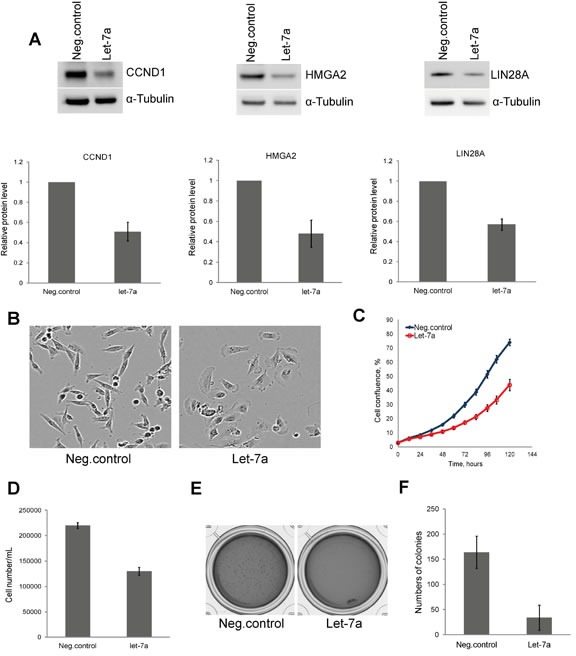
*Let-7a* represses cell proliferation and clonogenic capacity of MDA-MB-231 cells A, protein levels of CCND1, HMGA2, and LIN28A on day 3 post transfection and densitometric quantification of proteins normalized to α-tubulin, n=3, SD. B, phase-contrast images of cells on day 3 post transfection, magnification 10X. C, cell confluence-based growth curve. D, count of cells using Trypan Blue on day 3 post transfection, n=3, SD. E, colonies generated after 10 days of incubation in the methylcellulose-based medium. F, quantification of colonies by counting of MTT stained colonies, n=4, SD.

To assess the validity of our model system, we first investigated the protein level of three known *let-7* targets, *CCND1*, *HMGA2* and *LIN28A* upon *let-7a* transfection. Cells transfected with *let-7a* mimics (hereinafter *let-7a*) or with negative control oligos (hereinafter negative control) were lysed on day 3 post transfection and extracts were subjected to Western blot analysis. The levels of all three proteins were reduced to approximately 50% in *let-7a* transfected cells compared to the negative control (Fig. 1A). Interestingly, we observed that, in contrast to the spindle-shaped, mesenchymal-like morphology of untreated and negative control cells, the cells transfected with *let-7*a became more columnar and epithelial-like already 48 hours post transfection (Fig. 1B). Next, we investigated the effect of *let-7a* overexpression on cell growth. Cell growth was assessed by time-lapse live-cell imaging based on cellular confluence. Cells transfected with *let-7a* showed a time-dependent decrease in confluence compared to the negative control cells (Fig. 1C). The cell number and viability were assessed using Trypan Blue. As expected, *let-7a* significantly reduced the final number of cells in culture (Fig. 1D) with no effect on viability (Fig. S1A). Further analysis of caspase-3/7 activity to check for apoptosis revealed no difference between *let-7a* transfected and negative control cells (Fig. S1B). We performed colony formation assay to determine whether ectopic expression of *let-7a* impairs the clonogenic capability of MDA-MB-231 cells. The number of colonies generated from single cells was drastically reduced in *let-7a* treated samples (Fig. 1E and F), while the size of the colonies did not differ (Fig. S1C).

### *Let-7a* down-regulates key anabolic enzymes in MDA-MB-231 cells

**Figure 2:**
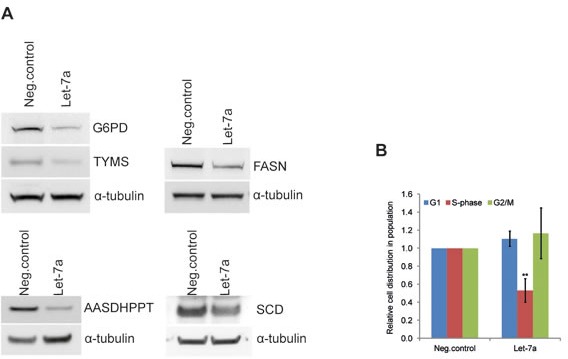
*Let-7a* affects key anabolic enzymes and cell-cycle progression in MDA-MB-231 A, Protein levels of G6PDH, TYMS, FASN, AASDHPPT, and SCD on day 3 post transfection. WBs quantification is shown in the Table 1. B, cell-cycle was analyzed by flow cytometry using Hoechst 33342 staining on day 3 post transfection, n=6, SD.

The effect of *let-7a* on global transcript levels was determined using microarray. Rank product analysis was employed to identify differentially expressed genes. The analysis revealed 873 probes, representing 754 genes, with significant changes in gene expression between *let-7*a transfected and negative control cells. Of these, 357 genes were down-regulated whereas 397 genes were up-regulated. Functional enrichment analysis of microarray data (GEne SeT AnaLysis-GESTALT-Toolkit) identified metabolic pathways to be highly enriched (Table S1). To investigate differences at the protein level we used SILAC-base proteome-wide analysis. We identified 37 proteins with significantly changed levels upon *let-7a* transfection. Of these, 27 proteins were down-regulated, and 10 proteins were up-regulated (Table S2). Comparison of the mRNA and protein changes revealed that 54% of the differentially expressed proteins were also changed in the same direction at the mRNA level (Fig. S2A). Enrichment analysis of changed proteins also showed metabolic pathways as the first group in the top ten list (Table S1). Among metabolic genes down-regulated by *let-7a* at the transcriptional and/or protein level we identified key enzymes of anabolic pathways, namely *G6PD*, inosine monophosphate dehydrogenase (*IMPDH2*), *FASN*, stearoyl-CoA desaturase (*SCD*) and 4-phosphopantetheinyl transferase (*AASDHPPT*) (Table 1). Protein level changes were further validated by Western blot analysis (Fig. 2 and Table 1). Differentially expressed mRNAs and proteins were compared with the list of predicted *let-7a* targets and revealed that among anabolic genes only SCD is a predicted *let-7* target (Fig. S2B). Since these enzymes are essential for macromolecular synthesis underlying cell cycle progression, we performed a cell-cycle analysis. The detection of Hoechst fluorescence by flow cytometry revealed that the fraction of cells in S-phase is reduced by 50% in *let-7a* transfected cells compared to the negative control (Fig. 2B).

**Table 1:** Expression of anabolic genes upon *let-7a* transfection

Gene name	Function	Microarray		SILAC		WB^1^
		FC	q-value	FC	q-value	FC
*IMPDH2*	De novo guanine nucleotide biosynthesis	−1.40	0.026	−1.41	0.053	n/a
*FASN*	Fatty acid synthase	−1.46	0.010	−1.15	0.461	−2.00
*TYMS*	Thymidin nucleotide biosynthesis	−1.29	0.113	−1.59	0.004	−2.66
*G6PD*	Pentose phosphate pathway (oxidative branch)	−2.08	0.000	−1.59	0.004	−3.44
*AASDHPPT*	4′-Phosphopantetheinyl transferase	−1.95	0.000	−1.79	0.000	−4.6
*SCD*	Stearol Co-A Desaturase	−1.66	0.000	n/a	n/a	n/a

FC = fold-change (negative of the inverse)

1 FC determined by densitometric analysis

### *Let-7a* regulates energy metabolism and mitochondrial ROS in MDA-MB-231 cells

**Figure 3:**
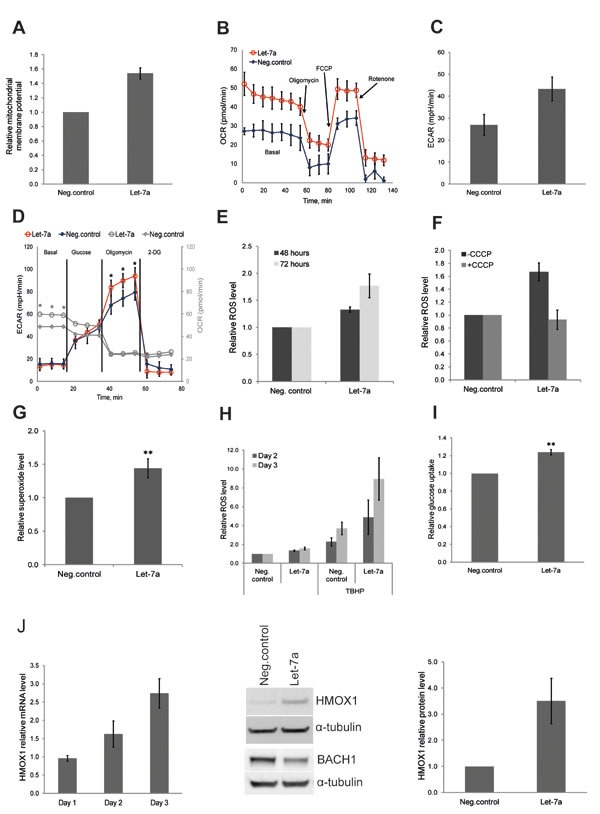
*Let-7a* regulates energy and redox metabolism in MDA-MB-231 cells A, mitochondrial membrane potential assessed by flow cytometry using JC1 dye on day 3 post transfection. Data are presented as a relative fold-change of JC1red/JC1green ratio, n=10, SEM. B, OCR under mito-stress test on day 3 post transfection, values are normalized to DNA content, n=4, SD. C, basal ECAR assessed in regular growth medium on day 3 post transfection, n=4, SD. D, ECAR under glyco-stress test on day 3 post transfection; OCR values are in gray. Values are normalized to DNA content, n=4, SD, **p<0.01. E, ROS levels assessed 48 and 72 hours post transfection. Values are compared to the negative control for each time point, n=5, SEM. F, ROS levels assessed on day 3 post transfection, with or without CCCP treatment, n=3, SEM. G, superoxide level assessed on day 3 post transfection by flow cytometry using MitoSOX, n=8, SD,**p<0.01. H, cells were treated with TBHP on day 2 and 3 post transfection 4 hours before ROS measurement. ROS levels in TBHP-treated cells were compared to the ROS levels detected in negative control without TBHP treatment, n=5, SEM. I, glucose uptake on day 3 post transfection using a fluorescent D-glucose analog, n=4, SEM, **p<0.01. J, mRNA level of *HMOX1* in *let-7a* transfected cells over time compared to the negative control cells. Protein level of HMOX1 and BACH1 on day 3 post transfection; densitometric quantification of HMOX1 normalized to α-tubulin, n=3, SD.

After we observed the effect of *let-7a* on key anabolic enzymes, we investigated whether *let-7a* also affects other metabolic functions, such as energy metabolism. Therefore, we examined mitochondrial membrane potential using JC-1, a mitochondria specific fluorescent probe that exhibits potential-dependent accumulation in mitochondria, with a concomitant shift in the peak of emission spectrum from green (≈529 nm) to red (≈590 nm). We found that the mitochondrial membrane potential was increased upon *let-7a* transfection (Fig. 3A), indicating enhanced mitochondrial activity. To confirm the specificity of the assay, we treated *let-7a* transfected cells or negative control cells with the mitochondrial uncoupler CCCP, which dissipates the proton gradient causing a drop of the electrochemical potential across the mitochondrial membrane. As expected, after CCCP treatment, no red fluorescence was detected in negative control or in *let-7a* treated cells, while the total mitochondrial content was unchanged, as shown by the green fluorescence (Fig. S3). To assess overall energy metabolism, we measured the oxygen consumption rate (OCR) and the extracellular acidification rate (ECAR) using the XF^e^ Extracellular flux Analyser. *Let-7a* increased basal respiration, but not spear respiratory capacity (Fig. 3B). Surprisingly, *let-7a* increased also basal ECAR (Fig. 3C). To investigate the maximal glycolytic capacity, we performed glycolysis stress test. Cells were incubated for 1 hour without glucose and pyruvate, and then in sequence glucose, oligomycin and 2-DG were added. The injection of oligomycin, which blocks ATP synthase, increased ECAR in *let-7a* cells significantly stronger than in negative control cells, indicating higher maximal glycolytic capacity (Fig. 3D, black and red lines). Of importance, even in the absence of glucose, OCR was higher in *let-7a* cells, suggesting that mitochondria utilize alternative carbon sources more efficiently (Fig. 3D, grey lines). Mitochondrial oxidative phosphorylation is the main source of ROS in a cell, and increased OXPHOS should lead to higher ROS production. Thus, we measured ROS level on day 2 and day 3 post transfection using 2′,7′-dichlorofluorescein diacetate (DCFDA). ROS level was increased up to approximately 2-fold in *let-7a* transfected cells, but not in negative control cells, in a time-dependent manner (Fig. 3E). It has been widely demonstrated that there is a positive correlation between mitochondrial membrane potential and ROS production [25, 26]. Moreover, mitochondrial ROS production is very sensitive to uncoupling [27]. To confirm that the detected increase in ROS is of mitochondrial origin, we pre-treated cells with the mitochondrial uncoupler CCCP which totally abolished the differences in ROS levels between *let-7a* transfected and negative control cells (Fig. 3F). To further validate the mitochondrial origin, we measured mitochondrial superoxide level using highly selective dye. We found mitochondrial superoxide increased in *let-7a* transfected cells compared to the negative control (Fig. 3G). Increased endogenous ROS level should sensitize cells to further oxidative stress. To assess that, we treated *let-7a* and negative control cells with the strong oxidant TBHP. ROS levels triggered by TBHP were significantly higher in *let-7a* cells than in negative control (Fig. 3H), and growth repression was stronger (Fig. S4). The acceleration of both glycolytic and OXPHOS pathways in *let-7a* transfected cells may require higher energy substrate influx and consumption. We thus measured glucose uptake in transfected cells using the fluorescent glucose analogue 2-NBDG. *Let-7a* treated cells displayed higher glucose uptake than negative control cells (Fig. 3I). The rate limiting glycolytic enzyme pyruvate kinase M2 (PKM2) can accelerate or slow down the glucose flux through glycolytic pathway by modulating its enzymatic activity. We measured the PKM2 activity and found that it was higher in *let-7a* transfected cells (Fig. S5).

Microarray analysis revealed that *let-7a* treatment induced antioxidant response genes in parallel with ROS (Table 2). Among them, heme oxygenase 1 (HMOX1) has previously been shown to be induced by the *let-7* family in human hepatocytes through the direct targeting of transcriptional repressor BACH1 [28]. We assessed the expression of these two genes and confirmed that HMOX1 is induced, while BACH1 is down-regulated by *let-7a* also in MDA-MB-231 breast cancer cells (Fig. 3J).

**Table 2:** Expression of antioxidant response genes upon *let-7a* transfection

Gene name	Function	FC	q-value
*MT1X*	Metal-binding protein	2.22	0.000
*MT2A*	Metal-binding protein	1.63	0.006
*MT1G*	Metal-binding protein	1.50	0.009
*MT1A*	Metal-binding protein	1.39	0.042
*SOD2*	Superoxide dismutase mitochondrial	1.53	0.008
*TXNRD1*	Thioredoxin reductase	1.69	0.008
*GSTM3*	Conjugation of reduced glutathione to exogenous and endogenous hydrophobic electrophiles	1.50	0.008
*CTH*	Converts cystathione derived from methionine into cysteine	1.51	0.008
*HMOX1*	Heme oxigenase	1.52	0.013
*FTH1*	Ferritin	1.62	0.017

FC = fold-change

### *Let-7a* regulates mitochondrial ROS in WM239 melanoma cells

**Figure 4:**
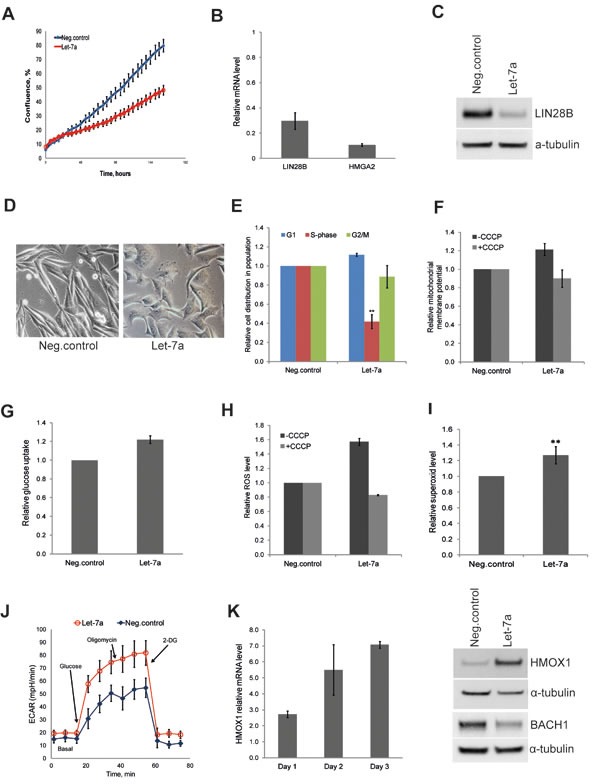
*Let-7a* affects proliferation and redox pathways in WM239 melanoma cells A, cell confluence-based growth curve. B, relative mRNA levels of *HMGA2* and *LIN28B* in *let-7a* transfected cells compared to the negative control, n=3, SD. C, LIN28B protein level in *let-7a* transfected cells. D, phase-contrast images of cells on day 3 post transfection, magnification 10X. E, cell-cycle was analyzed by flow cytometry using Hoechst 33342 staining on day 3 post transfection, n=4, SD. F, mitochondrial membrane potential on day 3 post transfection using JC1 dye with or without CCCP. CCCP-treated *let-7a* sample were normalized to the CCCP-treated negative control. Data are presented as a relative fold-change of JC1red/JC1green ratio, n=3, SEM. G, glucose uptake measured on day 3 post transfection using a fluorescent D-glucose analog, n=3, SEM. H, The histogram shows ROS level on day 3 post transfection with or without CCCP, n=3, SEM. I, The histogram shows superoxide level assessed on day 3 post transfection using MitoSOX, n=4, SD, **p<0.01. J, ECAR under glyco stress test on day 3 post transfection, n=4. K, mRNA level of HMOX1 in *let-7a* transfected cells over time compared to the negative control, n=3, SD. HMOX1 and BACH1 protein level on day 3 post transfection.

To extend our findings to a cancer type of non-epithelial origin, we investigated the effect of *let-7a* on the metastatic melanoma cell line WM239. The effect of *let-7a* on cell growth and target gene expression on day 3 post transfection confirmed effects observed in MDA-MB-231 cells (Fig. 4A, B and C). Interestingly, similarly to MDA-MB-231 cells, melanoma cells also displayed morphologic changes upon *let-7a* transfection; the cells became rounder and lost the protrusions (Fig. 4D). Cell-cycle analysis showed that *let-7a* strongly reduced number of cells in S-phase (Fig. 4E). We further investigated mitochondrial membrane potential and glucose uptake, and found both parameters higher in *let-7a* transfected cells than in negative control cells (Fig. 4F and G). Similarly, endogenous ROS was increased in *let-7a*, but not in negative control cells, and this increase was abolished by CCCP treatment (Fig. 4H). Using MitoSox we assessed that mitochondrial superoxide level is increased in *let-7a* overexpressing cells (Fig. 4I). However, contrary to the MDA-MB-231 cells, we did not detect significant differences in OCR between *let-7a* and negative control (Fig. S6). On the other hand, *let-7a* transfected melanoma cells displayed stronger increase in ECAR under glycolytic stress test (Fig. 4J), confirming the data obtained in MDA-MB-231. Western blot analysis showed that *let-7a* also in this cell line induced HMOX1 and repressed BACH1 (Fig. 4K).

### *Let-7a* sensitizes breast cancer and melanoma cells to doxorubicin

**Figure 5:**
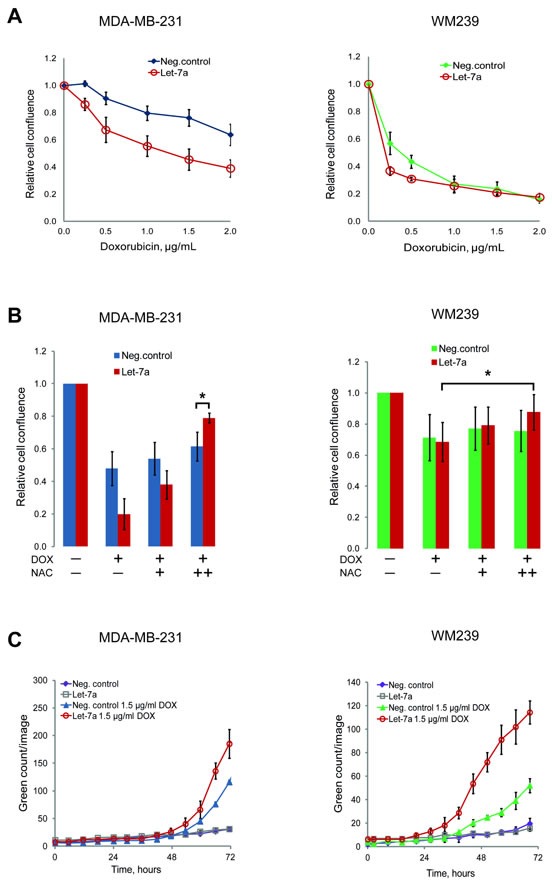
*Let-7a* sensitizes MDA-MB-231 and WM239 cells to doxorubicin A, cells transfected with *let-7a* or negative control treated with 5 different concentrations of doxorubicin for 72 hours. The cell confluence of doxorubicin treated cells was normalized to that of doxorubicin untreated cells and the ratio was reported on the graph against doxorubicin concentrations. n=5, SD. B, cells were pre-treated with 2.5 and 5 mM of NAC for 5 hours before adding 2 μg/mL of doxorubicin. Confluence of cells treated with only doxorubicin or with doxorubicin and NAC was normalized to the confluence of transfected cells without any treatment, n=5, *p<0.05. C, caspase 3/7 based apoptosis assay was performed on transfected cells with or without doxorubicin treatment. Apoptosis reagent was added 48 hours after transfection and the cells were followed in the Incucyte. Representative experiment is shown, n=3.

The anti-cancer drug doxorubicin has been shown to induce apoptosis through the generation of ROS [29]. However, this causes cytotoxicity in healthy tissues as well. Obtaining therapeutic efficacy at lower drug doses could reduce side-effects. We tested if the combination of doxorubicin with *let-7a* could be beneficial in this respect. Doxorubicin inhibited cell growth in both MDA-MB-231 and WM239 cells in a dose-dependent manner, but in cells transfected with *let-7a* the effect of doxorubicin at the lower concentrations was stronger than in negative control (Fig. 5A). We hypothesized that this occurs through the cumulative effect of ROS generated by *let-7a* and by doxorubicin. If so, an antioxidant treatment with NAC should abolish this effect. Pre-treatment of the cells with NAC attenuated the doxorubicin effect in both *let-7a* and negative control samples in a NAC concentration-dependent manner, with larger effect on *let-7a* transfected cells (Fig. 5B). Altogether, these data support the hypothesis that the increased sensitivity of *let-7a* treated cells to doxorubicin is caused by the *let-7a*-induced ROS. To assess if the reduction of cell number is due to the apoptosis, we performed the caspase 3/7-based assay. In melanoma cells *let-7a* strongly increased apoptotic death, while in MDA-MB-231 the difference compared to the negative control was not so pronounced (Fig. 5C).

## DISCUSSION

A distinctive metabolic feature of all rapidly dividing cells, including cancer cells, is the up-regulation of key anabolic enzymes. By analyzing the global transcriptome and proteome, we identified a number of key metabolic enzymes down-regulated in MDA-MB-231 cells upon *let-7a* transfection. Importantly, all of them fall into two major biosynthetic pathways: nucleotide (*G6PD*, *TYMS*, *IMPDH2*) and lipid (*FASN*, *AASDHPPT, SCD*) biosynthesis. G6PD is a rate-limiting enzyme of the oxidative branch of PPP that yields ribose 5-phosphate, a precursor for the *de novo* nucleotide biosynthesis, as well as the reducing equivalent NADPH, an essential cofactor of glutathione reductase. Inhibition of PPP causes NADPH depletion with a consequent decrease of the reduced-to-oxidized glutathione ratio [30]. A combination of reduced G6PD level and increased endogenous ROS should lead to the depletion of both NADPH and GSH. Surprisingly, we did not detect any reduction neither in NADPH level, nor in GSH (Fig. S8) in *let-7a* transfected cells, suggesting that alternative cellular sources may replenish the NADPH pool [31]. Both TYMS and IMPDH2 are up-regulated by the MYC oncoprotein [32, 33], which is a target of *let-7*, suggesting an indirect regulatory mechanism. TYMS is an essential enzyme of *de novo* thymidine monophosphate biosynthesis. Elevated TYMS level in cancer cells is considered to contribute to multidrug resistance. Of note, TYMS is a target of 5-fluorouracil, a chemotherapeutic drug used to treat a large variety of cancers [34]. IMPDH2 is a rate-limiting enzyme of the guanine nucleotide biosynthesis, also essential for DNA replication. The repression of these genes may be part of antiproliferative function of *let-7a*. A growing body of evidence indicates that *FASN*, the only enzyme that synthesizes fatty acids, acts as an oncogene and its overexpression is associated with poor prognosis in several malignant tumors [35, 36]. Thus, *FASN* has been considered as a target for anticancer therapy [37]. *AASDHPPT*, also reduced by *let-7a*, catalyzes the transfer of 4′-phosphopantetheine moiety to the acyl carrier domain of *FASN* [38]. This post-translational modification of the acyl carrier protein is essential for the functional maturation of *FASN*. The third enzyme of lipid biosynthesis pathway down-regulated by *let-7a* is a fatty acid desaturase *SCD*, which is also shown to be involved in cancer progression and considered to be an attractive target for anticancer therapy [39, 40]. The coordinated down-regulation of genes involved in nucleotide and lipid biosynthesis suggests a general repression of anabolic metabolism induced by *let-7* miRNA, which may contribute to its tumor suppressive function.

Mitochondrial ROS formation, as a consequence of ongoing OXPHOS process, is an important part of cellular metabolism and plays an essential role in cell physiology. The ROS species have for a long time been considered harmful by-products of mitochondrial respiration. However, there is growing evidence that ROS generated by a variety of cellular processes act as signaling molecules regulating a large number of different functions, from immune response to differentiation [41]. *Let-7* miRNAs promote a transition to terminal differentiation during development and maintain this differentiated state throughout life. In general, terminally differentiated cells rely on OXPHOS, while stem cells and cancer cells actively utilize aerobic glycolysis. Here we show that *let-7a* activates OXPHOS, which results in the generation of mitochondrial ROS in breast and melanoma cell lines. Even though the endogenous ROS level was elevated almost two-fold upon *let-7a* transfection, the cells did not display signs of apoptosis, suggesting that this ROS level was within a physiologic range. However, after treatment with the strong oxidant TBHP, *let-7a* transfected cells counteracted oxidative stress less efficiently than negative control cells did, likely due to cumulative effect of endogenous and exogenous ROS, which probably depleted their antioxidant defense capacity. Importantly, a recently published study has demonstrated that the epithelial-to-mesenchymal transition (EMT) of basal-like breast cancer is promoted by a metabolic switch to anaerobic glycolysis resulting in decreased ROS level [42]. MDA-MB-231 cells are classified as basal-like CD44^high^/CD24^low^ [43], and in these, even though *let-7a* accelerated glycolysis, it also promoted mitochondrial OXPHOS and ROS production, which usually is associated with a differentiated phenotype. Another study has reported that increased OXPHOS in cancer cells reduces cancer aggressiveness, while decreased mitochondrial metabolism contributes to cancer progression [44]. Our data support this concept by showing an inverse correlation between the OXPHOS and cancer aggressiveness, and suggest that *let-7* might counteract cancer-associated properties also at the metabolic level by promoting a more differentiated state.

The increase in endogenous ROS was associated with the coordinated up-regulation of antioxidant enzymes. Among them, the HMOX1 increase is of particular importance. In a recently published study, Hou W. *et al* reported that *let-7* induces HMOX1 by suppressing the transcriptional repressor BACH1 in human hepatocytes. Here we showed that *let-7a* overexpression induced HMOX1 and repressed BACH1 also in breast cancer and melanoma cell lines. HMOX1 catalyzes heme degradation to release ferrous iron, carbon monoxide (CO) and biliverdin. Biliverdin inhibits proliferative and angiogenic pathways in head and neck cancer, while CO at low concentrations has a potent anti-inflammatory effect [45, 46]. The induction of HMOX1 by *let-7* might potentiate anti-proliferative effects of *let-7* on cancer cells while decreasing inflammatory responses that often accompanies tumor development.

Interestingly, we also found increased ECAR as well as glucose uptake, indicating that *let-7a* enhances glycolytic flux. Previously, *let-7* has been shown to regulate glucose metabolism through the repression of mTOR pathway [23]. However, in breast cancer and melanoma cell lines examined in our study *let-7a* did not alter the phosphorylation status of mTOR nor of its downstream component S6 (Fig. S7A and B). This is in line with the recent study by Xiaoyu Ma *et al*., where they showed that in hepatocellular carcinoma *let-7* promoted OXPHOS without affecting mTOR pathway [24]. Multiple mechanisms governing the balance between glycolysis and OXPHOS might exist in different cancer types, which would reflect the metabolic heterogeneity and flexibility of cancer cells. The main function of upregulated glucose uptake and aerobic glycolysis in rapidly dividing cancer cells is to provide anabolic precursors. However, the repression of key anabolic enzymes and the decrease in proliferation in *let-7a* overexpressing cells is not compatible with acceleration of anabolic metabolism, suggesting an alternative role for the sustained glycolysis in these cells. Increase in ECAR has been seen during adipocyte differentiation [47]. As a promoter of terminal differentiation, *let-7a* might have triggered differentiation processes by similar mechanisms, which could explain the increase in the glycolytic rate. Furthermore, glycolysis is the main source of energy for cellular morphological transformations during differentiation [48, 49]. Dramatic shape changes observed in both cell lines upon *let-7a* transfection might therefore, at least partly, explain increased need for glycolysis.

To extend our findings to a cancer type different from the epithelial one, we overexpressed *let-7a* in the WM239 melanoma cell line. Although OCR was unchanged in *let-7a* transfected cells, we detected increased mitochondrial membrane potential and superoxide ROS level as well as glucose uptake, while a pre-treatment with CCCP abolished the ROS formation. Increased mitochondrial activity, but not the oxygen consumption might derive from defects in ETC complexes [50, 51]. *Let-7a* also induced the expression of HMOX1 and repressed BACH1 in WM239 cells. These data reinforce the hypothesis of the general biological role of *let-7* in regulating mitochondrial and redox metabolism.

Doxorubicin, a widely-used antitumor drug, blocks mitosis by specific intercalation within the DNA double helix. However, the treatment has severe side-effects, such as cardiomyopathy, making it imperative to obtain a therapeutic effect at the lowest dose possible. *Let-7* miRNA is often reduced in cancer cells and the restoration of its physiological levels is expected to have a therapeutic potential [52]. We investigated whether cancer cells can be sensitized to doxorubicin by increasing *let-7a* levels. A combination of *let-7a* overexpression and doxorubicin treatment produced a powerful effect on cell proliferation at 0.25 μg/mL of doxorubicin, the lowest dose tested, in both breast cancer and melanoma. The addition of antioxidant NAC attenuated this effect indicating that it occurred through the cumulative ROS level. In WM239 cells caspase-based apoptosis assay showed a striking increase in apoptotic cells in *let-7a* sample after the doxorubicin treatment, while in MDA-MB-231 cells the difference in apoptosis between *let-7a* treated and negative control cells was less prominent. The difference between cell lines might be explained by another type of ongoing cellular death, like necrosis, in MDA-MB-231 cells. In this study we uncovered a role of *let-7* miRNA in regulating energy and redox metabolism. Our data provide a *new insight* into the tumor suppressive mechanisms of *let-7* miRNA and offers new therapeutic opportunities for evaluation.

## MATERIALS AND METHODS

### Cell lines and culturing

The MDA-MB-231 triple-negative breast cancer cell line was obtained from American Type Culture Collection and the WM239 metastatic melanoma cell line from the Wistar Institute. Cell lines were STR-DNA profiled (Genotyping core facility, Oslo University Hospital) and are routinely mycoplasma tested. MDA-MB-231 cells were cultured in RPMI1640 and WM239 in DMEM, both supplemented with 10% fetal bovine serum (all from Sigma, Steinheim, Germany), 1% L-glutamate (GlutaMAX, Sigma) and 1% of penicillin G and streptomycin sulphate (Sigma). Cells were passaged twice a week in order to keep the cell confluence between 15 and 80%.

### Transient transfection

Transient transfection was performed using 18 nM of *let-7a* pre-miR^TM^ miRNA Precursors or Negative control #2 oligos (Ambion, Grand Island, USA) and the lipidic transfection agent Interferin (PolyPLUS, Illkirch, France) according to the manufacturer's protocol. For more details see Supplementary Material and Methods (SMM).

### Western blotting

Total protein lysate was generated on day 3 post transfection using 3% SDS lysis buffer. The protein concentration was determined by the Bio-Rad Protein Assay (Bio-Rad, Hercules, USA). Proteins of interest were detected by primary antibody followed by the appropriate secondary Ab (anti-rabbit or anti-mouse (Dako, Glostrup, Denmark)). Relative expression of the detected proteins normalized to α-tubulin was quantified using the Gene Tools densitometry software (Syngene, Cambridge, UK). Relative quantification is presented as a mean ± SD of at least three independent experiments. For more details and the list of Ab used see SMM.

### Quantitative real-time PCR

Isolation of total RNA and cDNA synthesis were performed using Cell-to-Ct kit for mRNA (Ambion), following the manufacturers protocol. Reaction mix for quantitative polymerase chain reaction (qPCR) was prepared using cDNA from 100 to 500 cells/well and TaqMan gene expression master mix (Ambion). The relative expression levels were determined using the comparative threshold cycle (2^−ΔΔCT^). Ct values were normalized to the endogenous internal control genes TBP or GAPDH which maintained stable expression levels through experiments. Normalized Ct values were presented relative to negative control oligos. Results are presented as a mean ± SD of at least three independent experiments. For more details and the list of primers used see SMM.

### Microarray analysis

Total RNA was isolated by TriReagent (Ambion), according to the manufacture's instruction. Global mRNA expression analysis of MDA-MB-231 cells on day 3 post transfection with *let-7a* mimic or negative control oligos was performed at the Oslo University Hospital Genomics Core Facility (http://oslo.genomics.no), using the Illumina HumanHT12 v4 Expression BeadChip (Illumina, San Diego, USA) according to the manufacturer's protocol. All analyses were performed with species filter set to human. For more details see SMM.

### Stable isotope labeling with amino acids in cell culture (SILAC)

SILAC was performed using Pierce^®^ SILAC Protein Quantification Kit (Thermo scientific, Waltham, USA). MDA-MB-231 cells were cultured for 4 days in SILAC RPMI1640 medium supplemented with 10% of dialyzed FBS and 50mg ^13^C_6_ L-lysine-2HCl/^13^C_6_^15^N_4_ L-arginine-HCl or L-lysine-2HCl/L-arginine-HCl according to the manufacturer's protocol. For detailed description see SMM.

### Cell cycle analysis

On day 3 post transfection cells were trypsinized and resuspended in culture medium. 2 μg/mL of Hoechst 33342 (Thermo Scientific) were added, and the cell suspension was incubated for 20 min at 37°C. 1×10^5^ cells per sample were analyzed by flow cytometry at excitation/emission = 346/497 nm on LSR II (Becton Dickinson, Franklin Lakes, USA). The data were processed by FlowJo Version 7.2.4 (Tree Star, Ashland, USA) and Dean-Jett-Fox model was applied. Results are presented as a mean fold-change ± SD of at least three independent experiments.

### Cell growth

Cell growth was monitored using the IncuCyte Kinetic Imaging System (Essen BioScience, Welwyn Garden City, UK) that estimates cell number based on confluence. Cells were scanned every second hour from day 1 to day 3 post transfection. Additionally, cells were counted on day 3 post transfection using Trypan Blue (Sigma) and an automated cell counter Countess (Invitrogen, Grand Island, USA). Cell viability was assessed on day 3 post transfection by Trypan Blue as well as by Caspase-Glo 3/7 luminescence-based Assay (Promega, Mannheim, Germany), according to the manufacturers protocol. All tests were performed in triplicates, and at least three independent experiments.

### Colony formation assay

On day 2 post transfection 800 cells/well mixed with Methylcellulose-based medium (MethoCult™ H4100 STEMCELL technologies, Grenoble, France) supplemented with 10% FBS, 1% L-glutamine and 60% DMEM, were seeded in 24-well low adhesion plates in triplicates. Cells were incubated at 37°C for 10-14 days and subsequently stained with 0.4 mg/mL of 3-(4,5-dimethylthiazol-2-yl)-2,5-diphenyltetrazolium bromide (MTT) for four hours (Life Technologies, Grand Island, USA). Colonies larger than 50 μm were detected and counted on the Gel Count (Oxford Optronix, Abingdon, UK). Quantification is presented as an average fold-change to negative control ± SD of at least three independent experiments.

### Oxygen consumption and extracellular acidification rate

Oxygen consumption rate (OCR) and extracellular acidification rate (ECAR) were measured with a XF^e^ Extracellular Flux Analyzer (Seahorse Bioscience, North Billerica, USA) according to the manufacturer's instructions. Cells were seeded in Seahorse plate on day 2 post transfection and cultured overnight to reach 80% confluence. One hour before the measurement, the culture medium was replaced with the cellular assay medium (Seahorse Bioscience) and incubated for 1 hour in a CO_2_-free incubator. The mito- and glyco stress-test were performed according to Seahorse protocols, with the final concentrations of 1μM FCCP, 1μM oligomycin and 1μM rotenone, and 10mM of glucose, 1μM of oligomycin and 100mM of 2-Deoxy-D-glucose, respectively. OCR and ECAR were normalized to the protein concentration and/or DNA content in each well at the end of the experiment. Results are presented as an average fold-change ± SD of at least three independent experiments.

### Glucose uptake

Glucose uptake was measured using a fluorescent glucose analogue 2-(*N*-(7-Nitrobenz-2-oxa-1,3-diazol-4-yl)Amino)-2-Deoxyglucose (2-NBDG) (Invitrogen). Briefly, on day 3 post transfection culture medium was replaced with fresh medium containing 50 μM of 2-NBDG. The cells were incubated for 2 hours at 37°C. 1×10^5^ cells per sample were analyzed by flow cytometry at excitation/emission = 465/540 nm on LSR II. The data were processed by FlowJo Version 7.2.4. Results are presented as a mean fold-change ± SEM of at least three independent experiments.

### Mitochondrial membrane potential

Mitochondrial membrane potential was measured using the JC-1 fluorescent dye (Invitrogen). On day 3 post transfection, cells were treated with 2 μM of JC-1 and incubated for 45 min at 37°C. As a control for JC-1 specificity, additional samples were co-treated with 10 μM of CCCP or DMSO only. 1×10^5^ cells per sample were analyzed using LSR-II(BD) flow cytometer with laser 488 and emission filter 525/50 nm for green signal and laser 561 and emission filter 582/15 for red signal. Data were processed by FlowJo v7.6.5. Signal from aggregates (red) and monomers (green) of JC-1 was normalized to negative control which was set at 1. Data are presented as ratio of red to green signal and a mean fold-change ± SEM of at least three independent experiments.

### Cellular ROS level

Cellular ROS level was assessed using 2′,7′-dichlorofluorescein diacetate (DCFDA) Assay Kit (Abcam, Cambrige, UK). On day 3 post transfection, cells were collected into flow cytometry tubes in culture medium supplemented with 20 μM DCFDA and incubated for 2 hours at 37°C. 50 μM of tert-butyl hydrogen peroxide (TBHP) was used as a ROS positive control according to the manufacturer's protocol. During incubation the cells were re-suspended periodically by shaking. To dissipate the mitochondrial proton gradient, 10 μM of CCCP or DMSO-only control was added to the cells together with DCFDA and incubated for 1.5 hour at 37°C. 1×10^5^ cells per sample were analyzed at excitation/emission = 485/535 nm. Results are presented as a mean ± SEM of at least three independent experiments. For superoxide detection MitoSOX fluorescent dye was used (Lifetechnology). On day 3 post transfection cells were trypsinized and resuspended in the culture medium with 5 μM of MitoSOX dye, incubated for 25 min and subjected to the flow cytometry. 1×10^5^ cells per sample were analyzed at excitation/emission = 510/580 nm.

### Doxorubicin experiment

1000 cells/well were seeded 48 hours post transfection in 96 well-plate in culture medium and allowed to adhere. Subsequently, doxorubicin (CAELYX) was added to the cells in five different concentrations: 0.25 μg/mL, 0.5 μg/mL, 1 μg/mL, 1.5 μg/mL and 2 μg/mL. Cells were monitored in the Incucyte to estimate the cell growth, n=5. For the NAC experiment, cells were pre-treated with 2.5 mM or 5 mM of NAC 2 hours before the addition of doxorubicin and followed in the Incucyte, n=5. The cell confluence of doxorubicin-treated cells was normalized to the cell confluence of untreated cells and obtained ratio was reported on the graph against doxorubicin concentration. All tests were performed in triplicates. Results are presented as a mean ± SD of at least three independent experiments. For the apoptosis assay, 2.5 μM of cell CellPlayer™ Kinetic Caspase-3/7 Apoptosis Assay Reagent (EssenBio) was added together with doxorubicin. Count of green events was performed in the Incucyte ZOOM over time, n=3.

## SUPPLEMENTARY MATERIAL, FIGURES AND TABLES


