# Supplementary material for: Metabolic reprogramming of metastatic breast cancer and melanoma by let-7a microRNA
Source: Oncotarget. 2014 Dec 29;6(4):2451–65. doi: 10.18632/oncotarget.3235 (PMC4385863; doi:10.18632/oncotarget.3235)
Supplement: Supplementary file 1 [file oncotarget-06-2451-s001.pdf]

Metabolic reprogramming of metastatic breast cancer and melanoma by *let-7a* microRNA

Supplementary Material

Cell lines and culturing

Cells were kept in culture for no more than 4 months before thawing new low passage stocks that were ID-profiled. MDA-MB-231 cells and WM239 cells were ID-profiled in 2011 and 2014, respectively.

Transient transfection

$8x10^5$ cells/well were seeded in a 12-well plate, allowed to adhere, transfected and incubated at 37°C over night. The medium was changed the next day, and the cells passaged at day 2 post transfection.

Western blotting

The composition lysis buffer: SDS 3%, Tris-HCl 1% (w/v), glycerol 14% (v/v), β-mercaptoethanol 6.98% (v/v), 1X protease inhibitor mixture (Complete Mini, Roche) and 1X phosphatase inhibitor mixture (PhosSTOP, Roche).

Equal amount of proteins per well was loaded on 4-12% gradient NuPAGE Bis-Tris gel (Invitrogen) in either MES or MOPs running buffer (Invitrogen) and transferred to 0.45 polyvinylidene fluoride (PVDF) membrane (Millipore) previously activated in methanol.

Ab name	Cat N	Company	Dilution
HMGA2	25810002	SDI	1:10000
CCND1	2926	Cell Signalling	1:2000
LIN28A	3978	Proteintech Group	1:1000
LIN28B	4196	Cell Signalling	1:1000
G6PD	8866	Cell Signalling	1:1000
FASN	610963	BD	1:250
AASDHPPT	sc-130931	Santa Cruz	1:200
SCD1	2438	Cell Signalling	1:1000
HMOX1	P249	Cell Signalling	1:1000
BACH1	14018-1-AP	Proteintech Group	1:1000

Antibody signal was visualized by chemiluminescence (SuperSignal West Dura, Pierce) and captured by charge-coupled device camera (Synoptic Group).

Quantitative real-time PCR

qPCR was performed using the 7900HT Fast Real-Time PCR System (Applied Biosystems) and TaqMan® Gene Expression Assays.

Primers name	Cat N	Company
HMGA2	00971725	Life Technologies
LIN28B	01013729	Life Technologies
HMOX1	01110250	Life Technologies
Let-7a	4427975	Life Technologies

Microarray

Expression values were annotated using the file Human HT-2_V4_O_RZ_15002878_B.logx from Illumina. The raw data is available through Gene Expression Omnibus (GEO GSE60326). Expression data was quantile normalized and log2 transformed in J-Express 2012 [1]. Differential gene expression analysis was performed using Rank-product analysis, with a q-value < 0.05 as a cut-off to identify differential expressed genes [2]. The final list of genes were further analysed using the web-based GEne SeT AnaLysis-GESTALT-Toolkit (http://www.webgestalt.org/) [3].

SILAC

Nano-LC/LTQ-Orbitrap mass spectrometry

Labeled or non-labeled cells were transfected with *let-7a* or negative control oligos. An incorporation test was applied before performing a Nano-LC/LTQ-Orbitrap mass spectrometry (MS). The cell lysates from each labeling, heavy and light, were mixed 1:1 and subjected to SDS-PAGE. Each Coomassie G-250 stained gel lane was cut into 12 slices and in-gel digested using 0.1 µg of trypsin in 25 µl of 50 mM ammonium bicarbonate, pH 7.8. After micropurification using µ-C18 ZipTips (Millipore, Oslo, Norway), the peptides were dried in a SpeedVac and dissolved in 10 µl 1% formic acid, 5% acetonitrile in water and subjected to MS. Half of the volume was injected into an Ultimate 3000 nanoLC system (Dionex, Sunnyvale CA, USA) connected to a linear quadrupole ion trap-orbitrap (LTQ-Orbitrap XL) mass spectrometer (ThermoScientific, Bremen, Germany) equipped with a nanoelectrospray ion source. For liquid chromatography separation, an Acclaim PepMap 100 column (C18, 3 µm beads, 100 Å, 75 µm inner diameter) (Dionex, Sunnyvale CA, USA) capillary of 50 cm bed length was used. The flow rate was 0.3 µL/ min, with a solvent gradient of 7 % B to 35 % B in 110 minutes. Solvent A was aqueous 0.1 % formic acid, whereas solvent B was aqueous 90 % acetonitrile in 0.1 % formic acid. The mass spectrometer was operated in the data-dependent mode to automatically switch between Orbitrap-MS and LTQ-MS/MS acquisition. Survey full scan MS spectra (from m/z 300 to 2,000) were acquired in the Orbitrap with the resolution R = 60,000 at m/z 400. The method used allowed the sequential isolation of up to the seven most intense ions for fragmentation on the linear ion trap using collision induced dissociation (CID) at a target value of 10,000 charges. Target ions already selected for

MS/MS were dynamically excluded for 60 sec. The lock mass option was enabled in MS mode for internal recalibration during the analysis. Other instrument parameters were set as previously described [4].

Protein identification and quantification

Protein identification and quantification were performed with MaxQuant [5] (v.1.2.2.5) utilizing the Andromeda search engine [6] with the IPI human database (v.3.68 – 87.061 human sequences). Trypsin was selected with up to two missed cleavage sites, and tolerance levels for identification were set to 10 ppm and 0.5 Da for MS and fragment MS/MS scans, respectively. In addition to heavy isotopes of arginine and lysine, methionine oxidation, deamidation of asparagines and glutamines, N-terminal protein acetylation and conversion of N-terminal peptide glutamine to pyro-glutamic acid were selected as variable modifications. We also included the reversed sequences as well as common contaminants into the database search, enabling estimation of the false discovery rate (FDR), which was set to 1% for reliable protein and peptide identification. For quantification, at least two quantification events were required per protein, and also the proteins were quantified in at least 2 of 3 replicates. All normalized protein ratios were subject to z-statistics for estimation of ratio significances, and a Benjamini-Hochberg corrected p-value < 0.10 was applied.

Senescence, PKM2 activity, GSH and NADPH assays

PKM2 activity, GSH and NADPH levels and senescence process were assessed by the respectives assays on day 3 post transfection according to the producer's protocol. All kits are from BioVision.

References

1. Dysvik, B. and I. Jonassen, *J-Express: exploring gene expression data using Java.* Bioinformatics, 2001. **17**(4): p. 369-70.
2. Breitling, R., et al., *Rank products: a simple, yet powerful, new method to detect differentially regulated genes in replicated microarray experiments.* FEBS Lett, 2004. **573**(1-3): p. 83-92.
3. Wang, J., et al., *WEB-based GEne SeT AnaLysis Toolkit (WebGestalt): update 2013.* Nucleic Acids Res, 2013. **41**(Web Server issue): p. W77-83.
4. Koehler, C.J., et al., *Isobaric peptide termini labeling for MS/MS-based quantitative proteomics.* J Proteome Res, 2009. **8**(9): p. 4333-41.
5. Cox, J. and M. Mann, *MaxQuant enables high peptide identification rates, individualized p.p.b.-range mass accuracies and proteome-wide protein quantification.* Nat Biotechnol, 2008. **26**(12): p. 1367-72.
6. Cox, J., et al., *Andromeda: a peptide search engine integrated into the MaxQuant environment.* J Proteome Res, 2011. **10**(4): p. 1794-805.

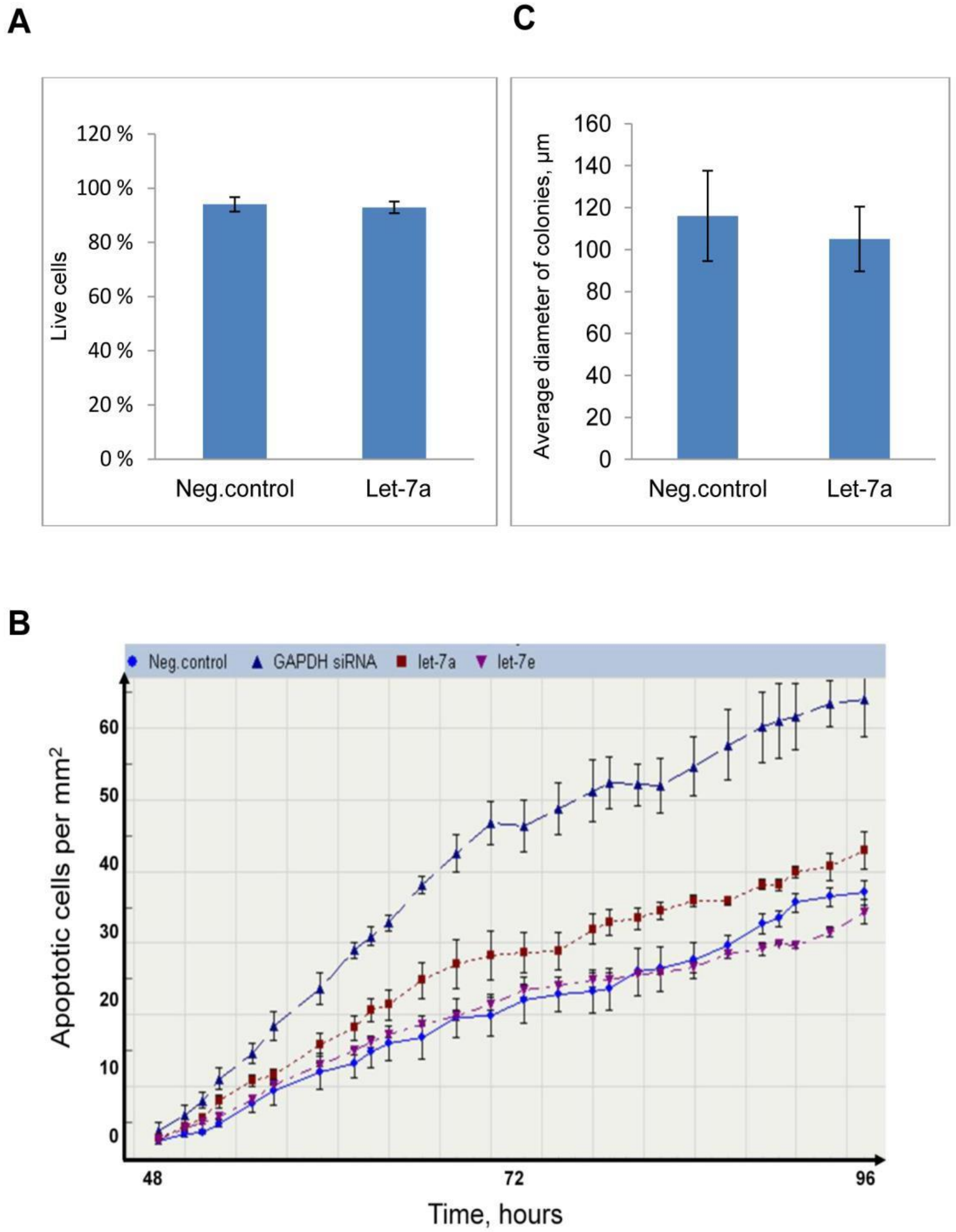

Figure S1. A, cells were counted on day 3 post transfection using Trypan Blue. The histogram shows the number of alive cells. Data are mean ± SD, n=3. **B**, Caspase 3/7 based apoptosis assay was performed on day 3 post transfection. GAPDH siRNA was used as a positive control. Representative experiment is shown. Bars are SEM. **C**, colony formation assay performed on day 3 post transfection. The histogram shows the diameter of colonies. Data are mean ± SD, n=4.

Table S1. Enriched KEGG pathways at transcript and protein levels.

Pathway	Genes[1]/reference genes[2]	p-value[3]
Down-regulated transcripts		
Metabolic pathways	32/1130	5.81E-11
Cell cycle	9/124	3.14E-07
N-Glycan biosynthesis	5/49	2.73E-05
TGF-beta signalling pathway	6/84	3.34E-05
Other types of O-glycan biosynthesis	4/46	0.0003
Up-regulated transcripts		
Metabolic pathways	33/1130	8.65E-11
Antigen processing and presentation	10/76	4.00E-10
Type I diabetes mellitus	8/43	1.33E-09
Rheumatoid arthritis	9/91	3.94E-08
NOD-like receptor signalling pathway	7/58	3.30E-07
Changed proteins		
Metabolic pathways	8/1130	4.26E-06
DNA replication	3/36	4.07E-06
Alanine, aspartate and glutamate metabolism	2/32	0.0003
Cell cycle	3/124	0.0002
Arginine and proline metabolism	2/54	0.001

1: the number of genes changed by *let-7a* in the pathway
2: the number of reference genes in the pathway
3: p-value from hypergeometric test

Table S2. Proteins changed upon *let-7a* transfection.

Down-regulated			Up-regulated		
	**FC**	**q-value**		**FC**	**q-value**
JUP	0.01	0.000	NAMPT	1.60	0.039
APOB	0.05	0.000	OAT	1.61	0.033
HBA1	0.09	0.000	DPYSL2	1.61	0.033
AASDHPPT	0.56	0.000	CLIC4	1.73	0.009
PDCD11	0.58	0.000	AADACL1	1.78	0.005
GSN	0.59	0.000	ALDH2	1.80	0.004
PGRMC1	0.59	0.000	ASNS	2.03	0.000
CPOX	0.60	0.001	SPANXB1	2.04	0.000
PRKAR2A	0.61	0.001	GFPT2	2.26	0.000
GMPR2	0.62	0.003	AKR1C3	2.55	0.000
TYMS	0.63	0.004			
G6PD	0.63	0.004			
ERO1L	0.64	0.005			
PABPC4	0.66	0.009			
TCERG1	0.66	0.012			
USP10	0.67	0.013			
TCOF1	0.67	0.016			
KIAA0664	0.67	0.016			
PREP	0.67	0.016			
MCM3	0.68	0.020			
MCM6	0.69	0.025			
IPO4	0.69	0.027			
NOL6	0.69	0.033			
PDE12	0.70	0.036			
MCM2	0.70	0.038			
MCM4	0.71	0.043			
SLC25A24	0.71	0.046			

FC = fold-change

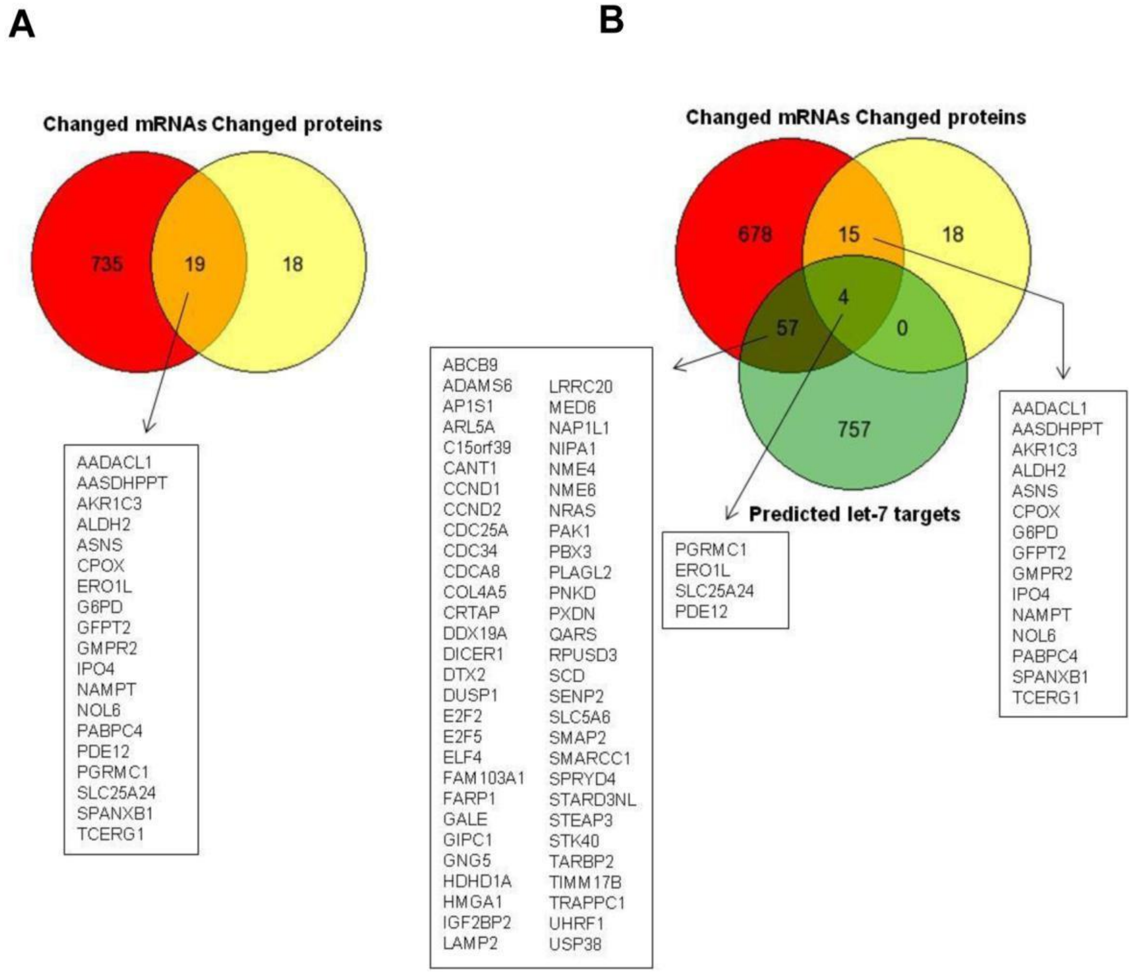

Figure S2. A, comparison of *let-7a* induced changes detected at the mRNA and the protein level detected by microarray (in red) and SILAC (in yellow), respectively, in MDA-MB-231 cells. **B**, comparison of *let-7a* induced changes with predicted *let-7* targets (in green) (TargetScan Human 6.2).

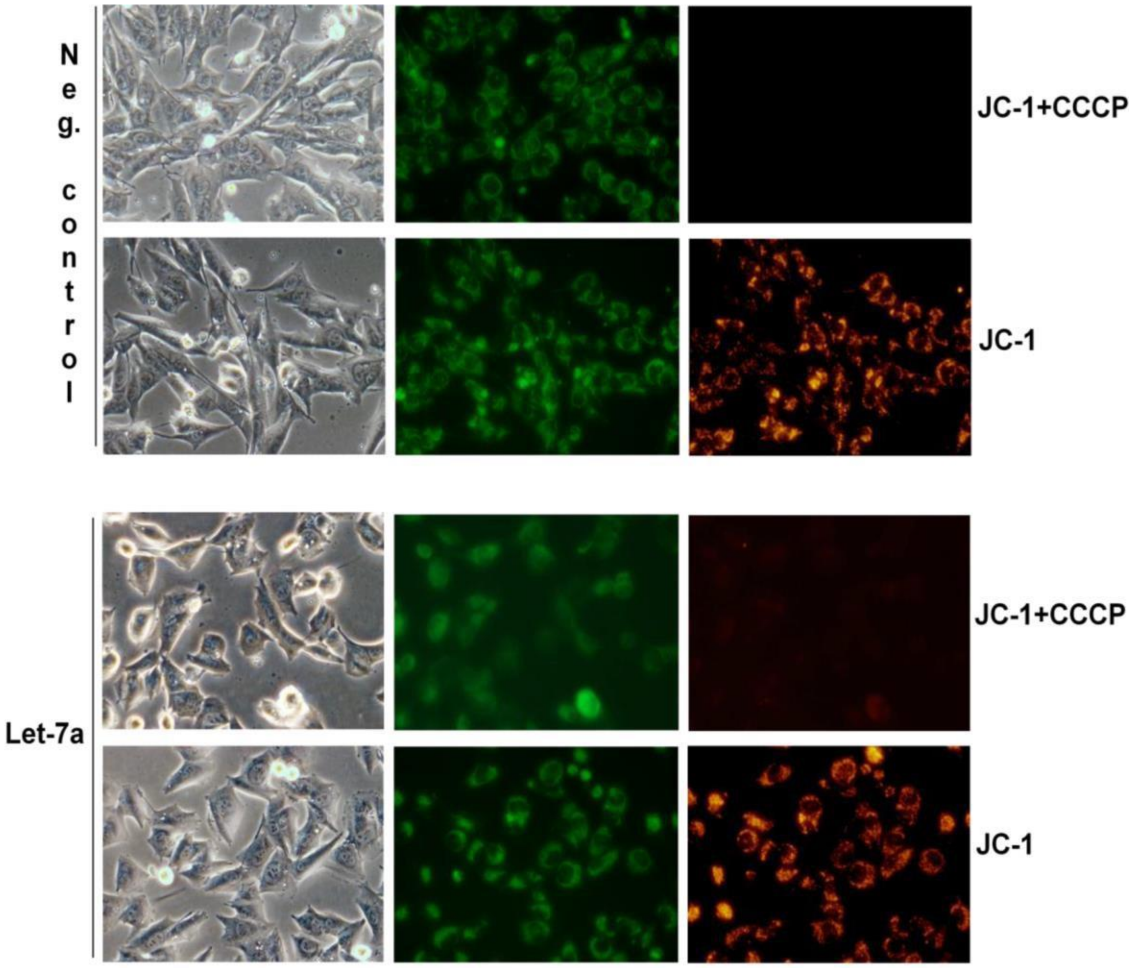

Figure S3. Detection of JC-1 dye red and green fluorescence by fluorescence microscopy in WM239 cells. *Let-7a* or negative control cells with and without CCCP treatment. After CCCP treatment no red fluorescence is detected, while green fluorescence is unchanged. Representative experiment is shown, n=3.

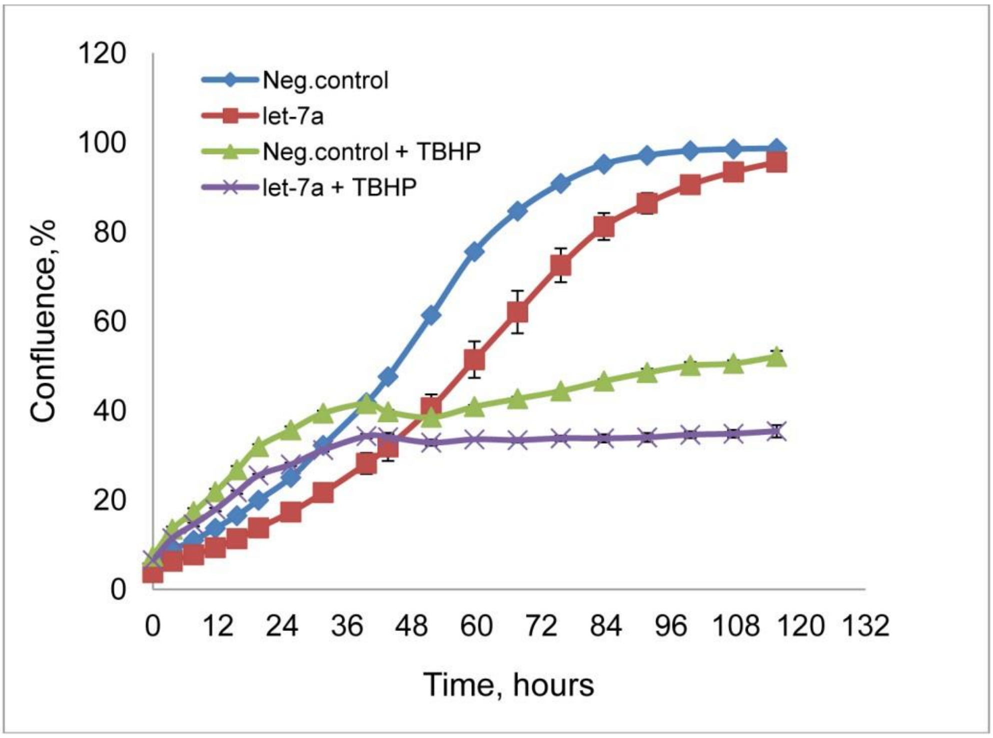

Figure S4. *Let-7a* overexpressing cells are more sensitive to oxidative stress. MDA-MB-231 cells were treated with TBHP on day 3 post transfection and cell growth followed in the Incucyte for the next 5 days. Representative experiment is shown, n=3.

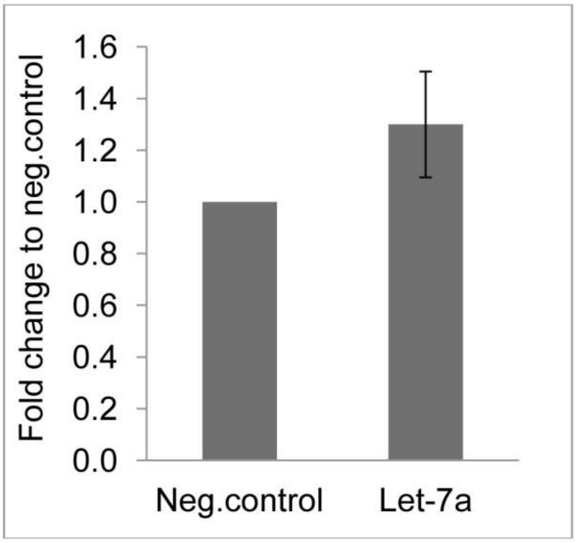

Figure S5. *Let-7a* overexpressing cells have increased PKM2 activity. Enzymatic activity of PKM was detected on day 3 post transfection using Pyruvate Kinase Activity Assay Kit (Biovision). Values were normalized to the cell confluence. Data are mean±SD, n=5

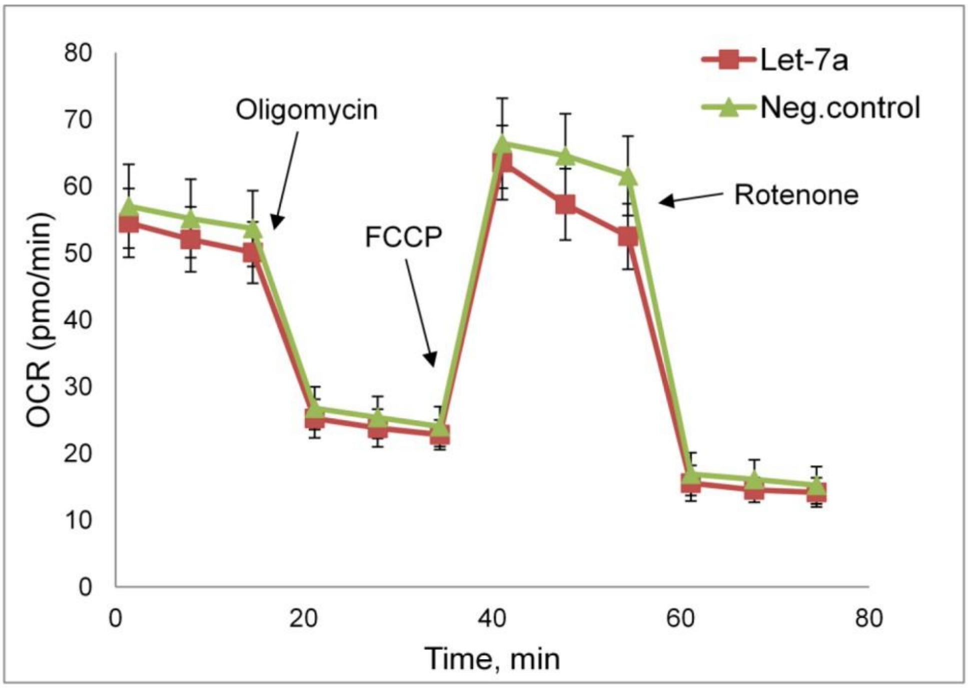

Figure S6. No changes in OCR were detected in WM239 cells upon let-7a overexpression. Cells were analyzed for OCR on day 3 post transfection using XF[e]96 Flow Analyzer. Values were normalized to the DNA content. Representative experiment is shown, n=4.

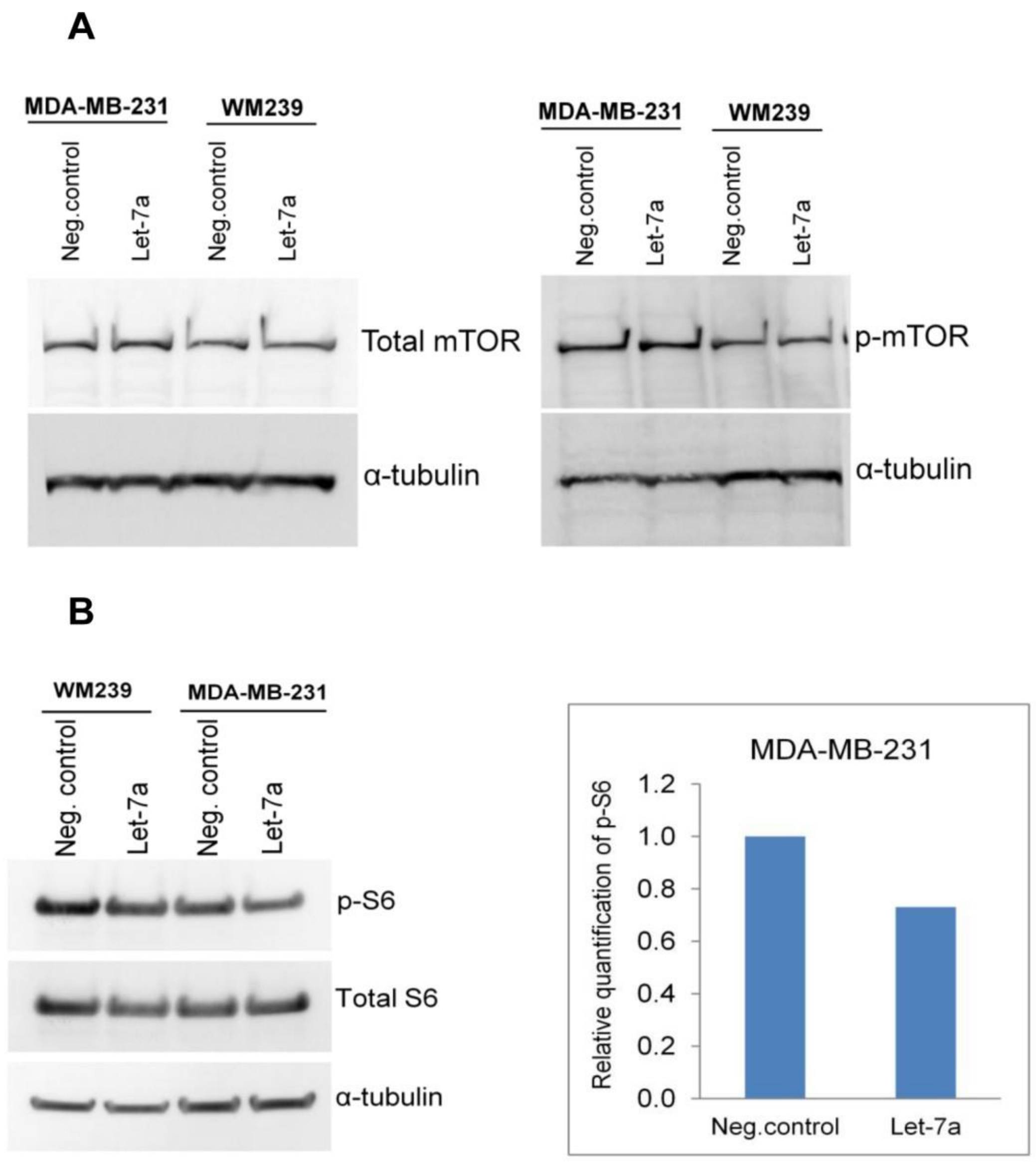

Figure S7. Western blot analysis of mTOR pathway components. **A**, mTOR protein level and phosphorylated mTOR level are unchanged in both cell lines on day 3 post transfection. **B**, phosphorylation of S6 is slightly reduced in MDA-MB-231 cells and unchanged in WM239 cells. The histogram shows protein quantification of p-S6 normalized to total S6 in MDA-MB-231 cells.

A

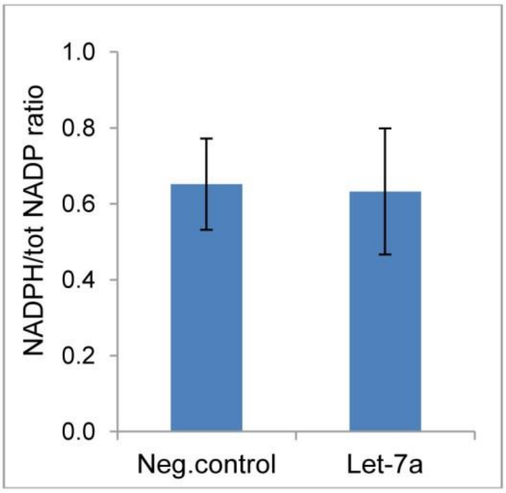

B

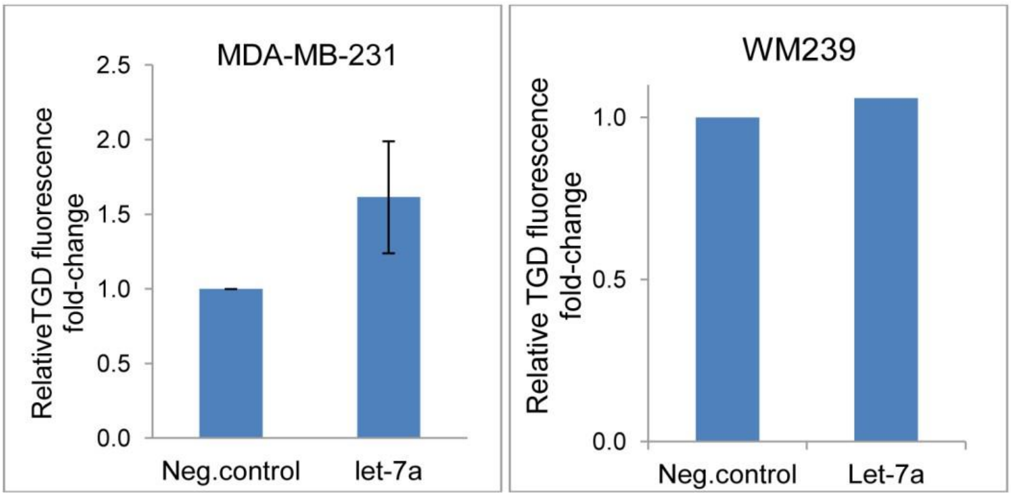

Figure S8. A, NADPH level measured in MDA-MB-231 cells on day 3 post transfection using colorimetric assay (Abcam). **B**, Level of the reduced form of glutathione detected on day 3 post transfection in WM239 and MDA-MB-231 cells by flow cytometry using Thiol Green Dye (Abcam)
